# Reducing the Digital Divide: Distribution of Technology to Increase Access to Pediatric Specialty Care via Telehealth During the COVID-19 Pandemic

**DOI:** 10.1089/tmr.2024.0087

**Published:** 2025-02-21

**Authors:** Brianne Gilkes, Anna Ditkoff Dorsey, Erin Jones, Christina E. Love, Kimberly Milla, Jennifer Crockett, Jacqueline Stone, Andrew T. Zabel

**Affiliations:** ^1^ Kennedy Krieger Institute, Baltimore, Maryland, USA.; ^2^ Johns Hopkins University School of Medicine, Baltimore, Maryland, USA.

**Keywords:** access, equity, telehealth, telemedicine

## Abstract

**Introduction:**

Increased use of telehealth technology during the COVID-19 pandemic helped reduce the impact of some barriers to health care access (e.g., geographical distance) while amplifying the negative impact of others (e.g., poor internet availability). This quality improvement project evaluated a program established at a specialized hospital for children and adolescents with neurodevelopmental disabilities to improve access of patients and families to technologies necessary for telehealth-based care.

**Methods:**

Our telehealth access and device distribution program utilized Federal Communications Commission funding to distribute 336 iPads and 279 Wi-Fi hotspots to 414 patients recommended for the program by their clinicians. An average of 1.6 years later, participants received a satisfaction survey via text or email.

**Results:**

The referred patient cohort had higher economic disadvantage (average Area Deprivation Index = 7.67) and more language diversity (16% Spanish) compared with hospital averages. About 27% (*n* = 112) of caregivers completed the satisfaction survey. Most respondents, including 92% of Spanish speakers, reported receiving instructions in their preferred language. Approximately 80% of caregivers stated that the devices enabled telehealth visits. Notably, device abandonment/disuse was considerable, with only 63% of iPads and 36% of Wi-Fi hotspots still in use an average of 1.6 years after device distribution.

**Discussion:**

Program efforts were largely successful in facilitating telehealth access via the dissemination of iPads and Wi-Fi hotspots to a patient subpopulation with economic disadvantage and language differences. Follow-up feedback from participants suggests that additional check-ins and device monitoring may be necessary to prevent device abandonment/disuse and maintain longevity of telehealth access.

## Introduction

In the U.S., the COVID-19 pandemic resulted in 104,137,196 confirmed cases and 1,125,366 deaths in the United States by 2023.^1^ In 2020, government-issued stay-at-home orders led many individuals to transition their work, school, and health care activities to virtual settings. Our specialty hospital rapidly transitioned to telehealth to maintain service provision to patients with neurodevelopmental disabilities who were at particularly elevated risk during the pandemic.^2^ While challenging, rapid adoption and/or expansion of telehealth-based care was essential to ensuring continuity of care for patients when in-person care was not possible or was potentially unsafe.

Patient and parental reception to the swift adoption/expansion of telehealth has generally been positive.^3^ However, concerns persist regarding health disparities, particularly among racial and ethnic groups’ access to technology.^4^ Race- and ethnicity-based differences in telehealth usage have been noted prior to and during the pandemic. White-Williams et al. (2023) found lower telehealth utilization among Hispanic patients compared with non-Hispanic White and Black patients both before and during COVID-19. Hispanic patients were 41% less likely to have had a telehealth appointment pre-pandemic. Although Hispanic patients’ telehealth usage rose during the pandemic, it remained below that of non-Hispanic White and Black patients. This suggests persistent barriers to telehealth adoption among this population despite increased provider utilization during the pandemic.^4^

Disparities in telehealth utilization often stem from inadequate internet access and technology availability. The pandemic spotlighted the digital divide, delineating those with access to information technology from those without.^5^ While virtual care surged amid the pandemic, access to the necessary internet, technology, and digital skills remained unequal. Approximately 42 million Americans lack high-speed internet.^6^ Moreover, there is inequity in access to digital devices with sufficient data for telehealth participation,^7^ especially notable in low-income or medically underserved populations.^8^ These groups often face greater barriers accessing the technology vital for telehealth appointments compared to more privileged populations.

Cultural factors can compound telehealth disparities. While telehealth addresses geographic barriers, it does not necessarily address differences in language and health care beliefs.^4^ Non-English speakers engage less in telehealth care, as evidenced by reduced visits among Spanish-speaking patients during the pandemic.^9^ Franciosi et al.^10^ noted a decrease in non-English speaking patients across specialties except pediatric nonsurgical care. Shahid et al.^7^ found that patients lacking English proficiency encountered significant technology hurdles, even among tech-savvy individuals. These findings underscore the challenges faced by linguistically diverse patients in accessing telehealth services.

In response to disparities, our hospital launched a pandemic-era device distribution program to improve telehealth access. This study assessed the program’s impact on disadvantaged groups’ telehealth accessibility. We gauged impact by demographic analysis of program participants to ensure devices reached those most in need. In addition, we evaluated satisfaction ratings regarding language support and device assistance. Lastly, we examined if device provision enhanced patient-reported telehealth access. These assessments aimed to determine the program’s efficacy in bridging telehealth gaps experienced by vulnerable populations.

## Methods

### Context

This was a single-center, retrospective cohort study examining the telehealth device distribution program at the Kennedy Krieger Institute. We followed the Revised Standards for Quality Improvement Reporting Excellence (SQUIRE 2.0) for the reporting of this study.^11^

### Setting

Kennedy Krieger Institute is primarily a children’s hospital (ages 21 and under) and provides healthcare delivery (inpatient, outpatient, and community), early childhood and K-12 educational programs, research, training, and advocacy, all dedicated to improving the lives of individuals with disorders of the developing nervous system. The Institute is based in Maryland but is world-renowned internationally. We currently see about 27,000 unique patients annually, who come from our 50 United States and 30 countries; this represents over 280,000 outpatient visits per year.

Before March 2020, telehealth services at Kennedy Krieger were limited to specific programs and comprised a small portion of total patient visits. With the onset of the pandemic, this drastically changed. In April 2020, 95% of outpatient visits (*n* = 23,591) were conducted via telehealth. Throughout the pandemic, telehealth and in-person visits gradually balanced, reaching a 50/50 split by June 2022. By 2024, onsite visits stabilized at 60%, with telehealth constituting 40% of visits. The hospital’s large array of behavioral health-focused services (e.g., social work, psychology, psychiatry) largely account for the continuation of robust telehealth services at the Institute, with approximately 70% of behavioral health services provided via telehealth. With over 27,000 telehealth visits in the first quarter of 2024, ensuring patient access to Wi-Fi and digital devices remains crucial to ongoing operations.

### Intervention

Increasing health equity was an important part of our hospital’s pandemic planning. To address this goal, we secured funding from the Federal Communications Commission for a telehealth access and device distribution program. This initiative provided iPads and Wi-Fi hotspots to households lacking necessary devices or internet connectivity for telehealth care. Cultural sensitivity was prioritized, with resources delivered in the preferred languages of patients’ caregivers. The program aimed to bridge the digital divide exacerbated by the pandemic, ensuring equitable access to telehealth services for all.

Kennedy Krieger established a process for identifying households in need of iPads or Wi-Fi for telehealth and distributing the devices. Device distribution was initiated in the early phase of the COVID-19 pandemic, at which time the vast majority of clinical services were already conducted via telehealth. Clinicians were made aware of the device availability and were encouraged to refer patients for devices if their telehealth patients had limited/disrupted internet access, no internet access (resulting in audio-only phone visits), or insufficient devices to participate in telehealth. Families signed loaner agreements outlining their responsibilities, including device use, return, and security. As all patients were minors, agreements were signed by parents or caregivers. Devices were mailed securely, with signatures upon receipt.

While the majority of patients seen by our hospital speak English, approximately 3% of our hospital’s patients list Spanish as their preferred language, constituting 67% of our non-English speaking patients. We expected a comparable number of telehealth access and device distribution program referrals for patients and families who preferred communication in Spanish. To help ensure that these patients/families could easily activate and use these devices, the device distribution process was designed to be multilingual with information/instructions provided in English and Spanish. Loaner agreement forms were translated and delivered via email and text for ease of completion. We also offered verbal agreements aided by interpreters if needed.

### Study of the intervention

A brief follow-up survey was sent to caregivers of patients in September 2022 to evaluate the level of satisfaction with the device(s) they received. Given that Kennedy Krieger is primarily a children’s hospital, this follow-up survey project was limited to patients ages 17 and under. Survey links were emailed to caregivers who had received at least one device in the initial device distribution period (6/1/20 through 8/25/22). Our data management approach facilitated the linkage of caregiver responses from the survey to demographic variables (e.g., language preference, insurance type, race, and ethnicity) extracted from the electronic medical record (Epic).

### Satisfaction survey

A 10- to 16-question (depending upon display logic) online satisfaction survey was disseminated via text message and email to 414 households of patients who had received an iPad, a Wi-Fi hotspot, or both devices. Following the initial satisfaction survey distribution, the survey was resent 1 week and 2 weeks later to those who had not yet completed it. The electronic platform that hosted the survey, Qualtrics, included a language selection option that allowed respondents to toggle between English and Spanish at any point during the survey.

### Area deprivation index

The area deprivation index (ADI)^12^ is a mapping tool created from publicly available data used to characterize a region’s relative socioeconomic conditions. The ADI provides information about the level of disadvantage experienced by residents of a given area down to the Census block-group level and consists of the theoretical domains of income, education, employment, and housing quality.^13^ Both state and national scores are available. State-level ADI scores are ranked in deciles from 1 to 10, with 1 being the least disadvantaged and 10 being the most disadvantaged. The ADI has been associated with a number of adverse health outcomes, including COVID-19 incidence and rehospitalization rates.^14,15^

### Analysis

We utilized descriptive statistics to report patient demographics. Both parametric and nonparametric statistics were used to compare those patients/caregivers who responded to the satisfaction survey and those who did not. One-sample *t*-test was used to determine if the sample of survey responders had a greater level of sociodemographic disadvantage (i.e., ADI) compared with the hospital average. Descriptive statistics were used to present caregiver responses to the individual questions from the satisfaction survey. Statistical analysis was performed in SPSS, Version 27.

## Results

Four hundred fourteen (414) total households were sent devices (Table 1). Socioeconomic indicators in this cohort consistently diverged from hospital averages and suggested increased economic disadvantage. The mean average of the available ADI scores for these household (*n* = 337, M = 7.67, SD = 2.35) was significantly higher (i.e., more disadvantaged) than the mean average of the hospital (M *=* 4.92) *t*(336) = 21.4, *p* < 0.001. Households in this cohort had a higher percentage of public insurance (84%) compared with the hospital average (39%). Close to 16% of households in this cohort included a patient or parent who spoke Spanish, which was higher than the hospital average of 3%. Finally, the two programs that referred the greatest number of patients for devices (15.7% and 12.6%, respectively) were also the two programs at the hospital with the lowest rates of activation of patient portals (i.e., Epic MyChart) to electronic health records. This is noteworthy, as use of patient portals has been strongly associated with broadband access.^16^

**Table 1.** Demographic Characteristics of Households Receiving iPad and Hotspot Devices

	Total sample (*N* = 414)	Portion of sample who did not complete survey or provided incomplete responses (*n* = 302)	Portion of sample completing satisfaction survey (*n* = 112)
	*n* (%)	*n* (%)	*n* (%)
Device sent			
iPad alone	135 (32.6%)	103 (34.1%)	32 (28.6%)
hotspot alone	78 (18.8%)	58 (19.2%)	20 (17.9%)
iPad and hotspot	201 (48.6%)	141 (46.7%)	60 (53.5%)
Race			
Black or African American	207	148	59
Hispanic	35	23	12
Multiracial	17	14	3
Other	27	17	10
Other Asian	3	3	0
Unknown	62	53	9
White	63	44	19
Ethnicity			
*Unspecified	2	2	0
Not Hispanic, Latino/a, or Spanish origin	165	113	52
Other Hispanic, Latino/a, or Spanish origin	64	38	26
Unknown	183	149	34
Sex			
Female	126	94	32
Male	263	185	78
Unknown insurance	25	2	23
Private	29 (7.0%)	19 (6.3%)	10 (8.9%)
Public	347 (83.8%)	249 (82.7%)	98 (87.5%)
None	14 (3.4%)	12 (4%)	2 (1.8%)
Unknown	21 (5.1%)	19 (6.3%)	2 (1.8%)
Other	2 (0.5%)	2 (0.7%)	0 (0%)
Language			
English	348 (84.2%)	261 (86.4%)	87 (77.7%)
Spanish	65 (15.8%)	41 (13.6%)	25 (22.3%)
Area deprivation index			
Mean (SD)	M = 7.67 (2.35)	M = 7.71 (2.243)	M = 7.57 (2.62)

Note. The Area Deprivation Index ranges from 1 to 10 with 10 representing the most disadvantaged situations.

Each of the 414 households was sent the satisfaction survey questionnaire link via email and text message. One-hundred 29 households provided some information on the questionnaire (31% response rate), and 112 completed it (27% complete response rate). There were no significant differences noted in the ADI score when the 112 households that provided complete ratings were compared with those that did not provide ratings or provided incomplete ratings (*t*[335] = 0.37, *p* = 0.71). However, the percentage of households with a Spanish-speaking member was higher in the group that provided complete responses (22.3%) compared to the group that provided incomplete or no responses (13%) X^2^ (1, *N* = 413) = 6.70, *p =* 0.01). Descriptive and demographic information for the households who completed the satisfaction survey are presented in Table 1.

Survey responses were provided by caregivers (*n* = 108) or other family members an average of 607 days (SD = 199.4 days) after the devices were shipped to them. Most of the questionnaire respondents (95%) indicated they received instructions for the Wi-Fi hotspots or iPads in their preferred language. Ninety-two (92) percent of the Spanish-speaking households (*n* = 32) indicated they received instructions in their preferred language.

Among the 92 respondents who had been sent iPads, 88 (96%) gave responses indicating that they had received it. Of those, 84 (96%) iPads had been used at least once, and 55 (63%) iPads were still being used when the survey was completed an average of 1.6 years later. Among the 80 respondents who were sent Wi-Fi hotspots, 50 (63%) gave responses indicating that they had received it. Of those, 37 (74%) Wi-Fi hotspots had been used at least once, and 18 (36%) were still being used.

In general, respondents indicated a high level of satisfaction with the telehealth access and device distribution program. For respondents who indicated that they had received the devices, the majority “agreed” or “strongly agreed” that the instructions provided were helpful (91% iPad; 89% hotspot), it was easy to use the device (84% iPad; 82% hotspot), the device made it possible to have telehealth visits (82% iPad; 80% hotspot), and the device improved the quality of telehealth visits (82% iPad; 72% hotspot). Isolated comments about the iPads (*n* = 4) revealed several families had trouble setting them up. Isolated comments about the Wi-Fi hotspots (*n* = 12) indicated that several families had difficulty turning the devices on, getting them set up, or did not need them as they had access to their home’s Wi-Fi.

## Discussion

The rapid adoption of telehealth during the COVID-19 pandemic aimed to sustain health care access. However, concerns arose regarding disparities in digital access, particularly for economically disadvantaged and linguistically diverse patients. Our hospital initiated a telehealth access and device distribution program to address these challenges. This project demonstrated that distributing internet devices can bolster telehealth access for vulnerable populations. The majority of survey respondents reported that iPads (82%) and internet hotspots (80%) facilitated telehealth appointments, underscoring the program’s effectiveness in supporting equitable health care access.

There are several general findings that have been important for our ongoing efforts to maintain and expand telehealth access and to address barriers to this type of service delivery. First, this study revealed that clinician referral of patients to the telehealth access and device distribution program resulted in a referral sample with a greater level of economic disadvantage than our hospital average. As noted, patients and families were identified for inclusion in the telehealth access and device distribution program based upon clinician referral (for established patients) or administrative intake referral (for new patients). Clinician and/or administrative referral, rather than patient/self-referral, to the telehealth access and device distribution program was utilized as an initial means of managing demand and distribution of this limited resource. At the end of this project, it was clear that this referral and distribution method had resulted in the intended goal of delivering the devices to a particularly disadvantaged population. However, while clinician and administrative staff referral resulted in an appropriate cohort of patients/families for device distribution, it remains highly likely that there are patients/families with limited internet access who were not identified by this program selection method. As we have continued to develop the program, we have explored other identification methods that may expand access, including program recruitment via self-referral of patients/families who have participated in telehealth visits in the past. Additional inquiry will be needed to determine if self-referral results in a cohort of participants with a comparable level of economic disadvantage.

Second, clinician and/or administrative referral to our telehealth access and device distribution program resulted in a notable inclusion of non-English speakers (16%), surpassing expectations. From inception, program materials were available in English and Spanish, fostering better inclusion and satisfaction among non-English-speaking families. Most Spanish-speaking respondents indicated their language preferences were respected in program materials. In these ways, our patient identification and device distribution methodology helped meet aspirational standards that diverse, marginalized, and underrepresented populations do not experience telehealth inequity.^17^ Yet, further steps are warranted. Providing Spanish-language technical support and pre-recorded video demonstrations could aid parents with low literacy. Expanding material translation to other languages represented in our non-English-speaking patient populations, such as Arabic and Mandarin, would enhance accessibility and align with our commitment to equitable telehealth access. Finally, utilization of additional terms and explanations may be useful when working with multiple languages and translations. For instance, we utilized the Spanish phrase “punto de acceso” for hotspot in the Spanish translation of the satisfaction survey. Based upon feedback provided by several families, we suspect that this term may have been confusing to some caregivers, particularly those of who were sent two bundled devices (i.e., iPad and internet hotspot), but in the satisfaction survey only recollected receiving the iPad.

Third, we found that ongoing monitoring and caregiver contact may be necessary to ensure continuous device health and readiness for use. Our follow-up data suggests a degree of device abandonment/disuse that occurred in the months and years following distribution. In the absence of routine check-in data, it is difficult to determine the causes of these findings. There are several possible reasons, however, that may account for device abandonment/disuse that could be considered in similar future projects.

1. Once the pandemic subsided and onsite care resumed, patients/families might have discontinued device usage. While telehealth received positive feedback during the pandemic, some groups (e.g., parents of children under five; parents of children receiving physical and occupational therapy programs) showed less enthusiasm for ongoing telehealth compared with others.^3^ Families from these groups likely transitioned away from telehealth swiftly when onsite services became available.
2. Although families were provided with a helpline they could contact if they had questions, this program did not include a plan for regular follow-up contact with families once the devices were mailed. It is possible some of the devices were not activated, connected to the internet, or used for telehealth access. In addition, families may have stopped using their devices because of technical issues. More frequent and/or intentional check-in visits with participants to identify any potential concerns or issues with the devices may help to reduce the number of such scenarios.
3. Device restrictions, such as organizational management of iPads, may have led to disuse. For instance, while being used by parents/patients, the iPads continued to be managed through our organization. Organizational device management prevented the downloading of additional apps without organizational support and permission. As such, use of the devices was closely linked with ongoing involvement with telehealth services, and device use may have declined once the patient’s treatment course ended. Moving forward, permitting more multifunctional use of the devices for games, education, communication, etc., may help sustain the devices availability for future telehealth services.

While the present study used a survey to provide contextual understanding of device use and potential impact on telehealth care, there are a few avenues for future study that should also be considered. For example, the assignment of a language access coordinator to cohorts of device recipients may help provide a more direct way for non-English-speaking families to access care more easily while also providing more direct data regarding device receipt and use. There are also other ways that device use and impact could potentially be assessed, such as leveraging metadata related to the device itself (e.g., activation, signal-based use information) to better understand if and how often devices are being used. It may also be helpful to incorporate a simple phone call to device recipients to confirm device receipt and activation. However, there are ethical considerations that must be accounted for to avoid causing a perception of external monitoring within a disadvantaged population that may already be distrustful of the medical community; the access provided by these devices is intended to be helpful and noninvasive.

There are also several additional limitations that merit discussion, particularly the length of time between the device distribution and the dissemination of the satisfaction survey. Those patients who received devices in the earliest distribution may have received the satisfaction survey close to 2 years later. It is certainly possible that the duration of time may have biased the caregivers’ recollection of the usefulness of the devices (either negatively or positively) and/or may have interfered in their recall of having received specific devices (i.e., hotspots) at all. As such, we recommend shorter durations between intervention and assessment of satisfaction in future studies.

In summary, through its dissemination of iPads and Wi-Fi hotspots to families in need, the telehealth access and device distribution program was successful in expanding access to telehealth care. Several proposals have been made for refinement of this type of program, particularly regular and intentional follow-up contact to support the effective use of devices. The COVID-19 pandemic increased demand for telehealth-based services; patient satisfaction with those services ensured that telehealth continued after the pandemic ended. As telehealth endures and expands as a service model, ongoing attention to inequities in health care, including access to technology, will be necessary to provide or maintain access for our most vulnerable populations.

## Abbreviations Used

ADI: Area Deprivation Index
COVID-19: Coronavirus Disease 2019
Epic: Electronic Medical Record System
FCC: Federal Communications Commission
SPSS: Statistical Package for the Social Sciences
SQUIRE 2.0: Standards for Quality Improvement Reporting Excellence
U.S.: United States
